# Cardiac Resynchronization Therapy for Pacing-Related Dysfunction Post Cardiac Surgery in Neonates

**DOI:** 10.1016/j.atssr.2024.05.007

**Published:** 2024-06-05

**Authors:** Jesus C. Jaile, Jacquelyn D. Brady, Patrick Nelson, Wesam Sourour, Melvin C. Almodovar, Scott Macicek, Timothy W. Pettitt, Frank A. Pigula

**Affiliations:** 1 Department of Pediatric Cardiology, Children’s Hospital of New Orleans, New Orleans, Louisiana; 2 Department of Pediatric Cardiothoracic Surgery, Children’s Hospital of New Orleans, New Orleans, Louisiana

## Abstract

An infant with DiGeorge syndrome, multiple comorbidities, and truncus arteriosus type II underwent repair complicated by heart block necessitating placement of a dual-chamber bipolar pacing system with right ventricular leads and subsequent resynchronization with placement of left ventricular apical pacing leads. Resynchronization therapy improved QRS duration from 180 ms to 100 ms and ejection fraction from 25% to 54% over the course of 4 weeks with gradual return to normal function and eventual discharge.

The role of cardiac resynchronization therapy (CRT) following heart surgery in neonates is poorly defined. We present a term infant born with DiGeorge syndrome and truncus arteriosus type II, whose surgical repair was complicated by complete heart block, necessitating placement of a dual-chamber bipolar pacing system with right ventricular leads. Subsequently the patient developed severe left ventricular (LV) dysfunction, and, after residual anatomic or hemodynamic lesions were ruled out, CRT was performed with placement of LV apical pacing leads at 3 months of age.

A term infant with a postnatal diagnosis of truncus arteriosus type II, quadricuspid truncal valve, moderate truncal valve stenosis, and severe regurgitation was taken to the operating room on day 4 of life for ventricular septal defect closure, right ventricle-to-pulmonary homograft, and truncal valve repair consisting of resection of the smallest most dysplastic leaflet, creating a tricuspid truncal valve.

Postoperatively, the patient demonstrated a complete heart block (CHB) and underwent bipolar dual-chamber epicardial lead placement on day 12. The ventricular leads were placed anteriorly on the right ventricle near the diaphragm. Over 4 months postoperatively, the patient demonstrated a gradual decline in LV function associated with progressive dilatation and an increase in respiratory and inotropic support. Following initial truncus repair, the patient had low-normal LV systolic function with paradoxical intraventricular septal motion, progressing to severe with an ejection fraction (EF) of 23% prior to resynchronization (Supplemental Video 1). Over that period, the patient also exhibited a wide complex rhythm with QRS duration ranging from 180 to 200 ms, inappropriate axis, and intermittent loss of capture (Figure 1).Cardiac catheterization revealed no anatomic or physiologic explanation for progression of dysfunction.

**Figure 1:**
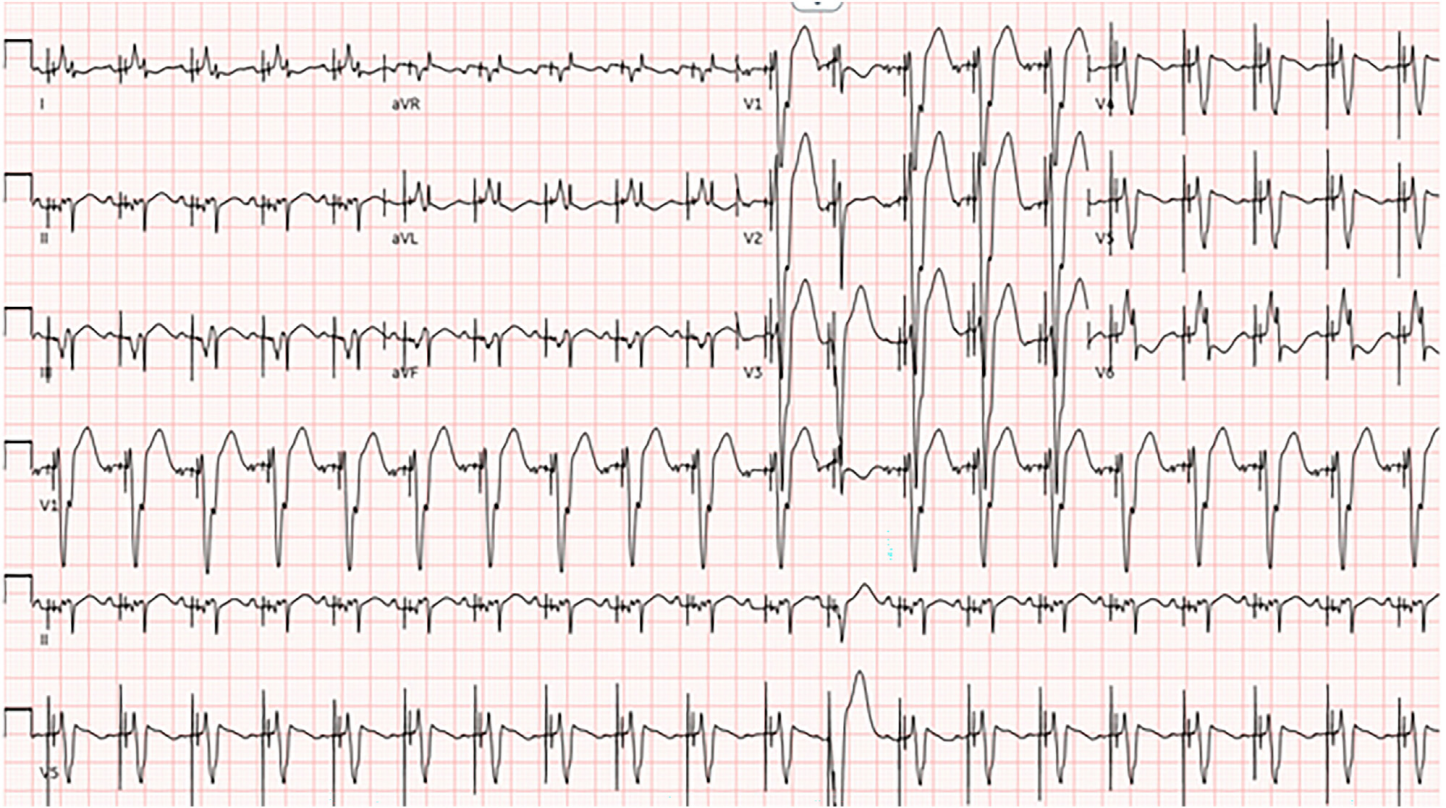
Electrocardiogram prior to resynchronization therapy, revealing an atrial-sensed ventricular paced rhythm with a wide complex QRS complex, duration of 202 ms, and inappropriate axis.

Based on these findings, the patient underwent CRT at 4 months of age. Bipolar steroid eluting leads were placed via left thoracotomy near the LV apex and tunneled to the upgraded pacemaker. Immediately following placement of the LV apical leads, QRS duration decreased to 114 ms, capture improved, and axis normalized (Figure 2).

**Figure 2:**
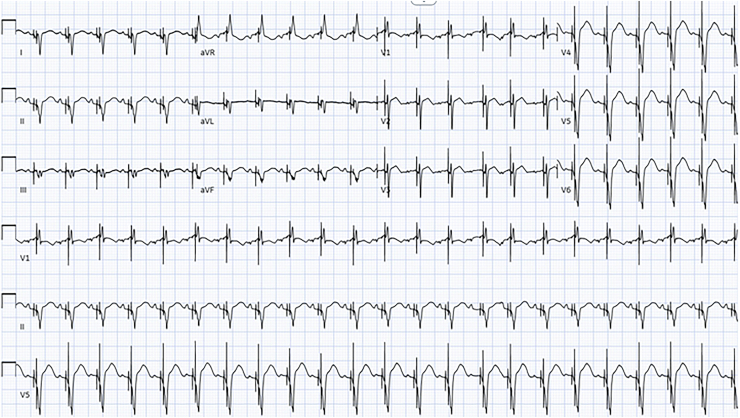
Electrocardiogram immediately after resynchronization therapy, revealing an atrial-sensed biventricular paced rhythm. The QRS duration narrowed to 114 ms with normal capture, PR interval, and axis.

Further synchronization was achieved by testing the programmable offset at different timing intervals. With an offset of 0 ms, QRS duration was 125 ms, and when the timing delay was increased to 80 ms, QRS duration increased to 151 ms (Figure 3A). Timing delay was then set to 40 ms, yielding a narrow QRS complex measuring 100 ms, and the decision was made to maintain those settings (Figure 3B). Immediate improvement in LV function was seen by transesophageal echocardiography in the operating room and, by 2 months postoperatively, LV function normalized (54% EF) by Simpson’s and biplane measurements. Subsequently, LV dimensions regressed, septal dyskinesia as well as free wall movement improved (Supplemental Video 2), and hemodynamics normalized.

**Figure 3:**
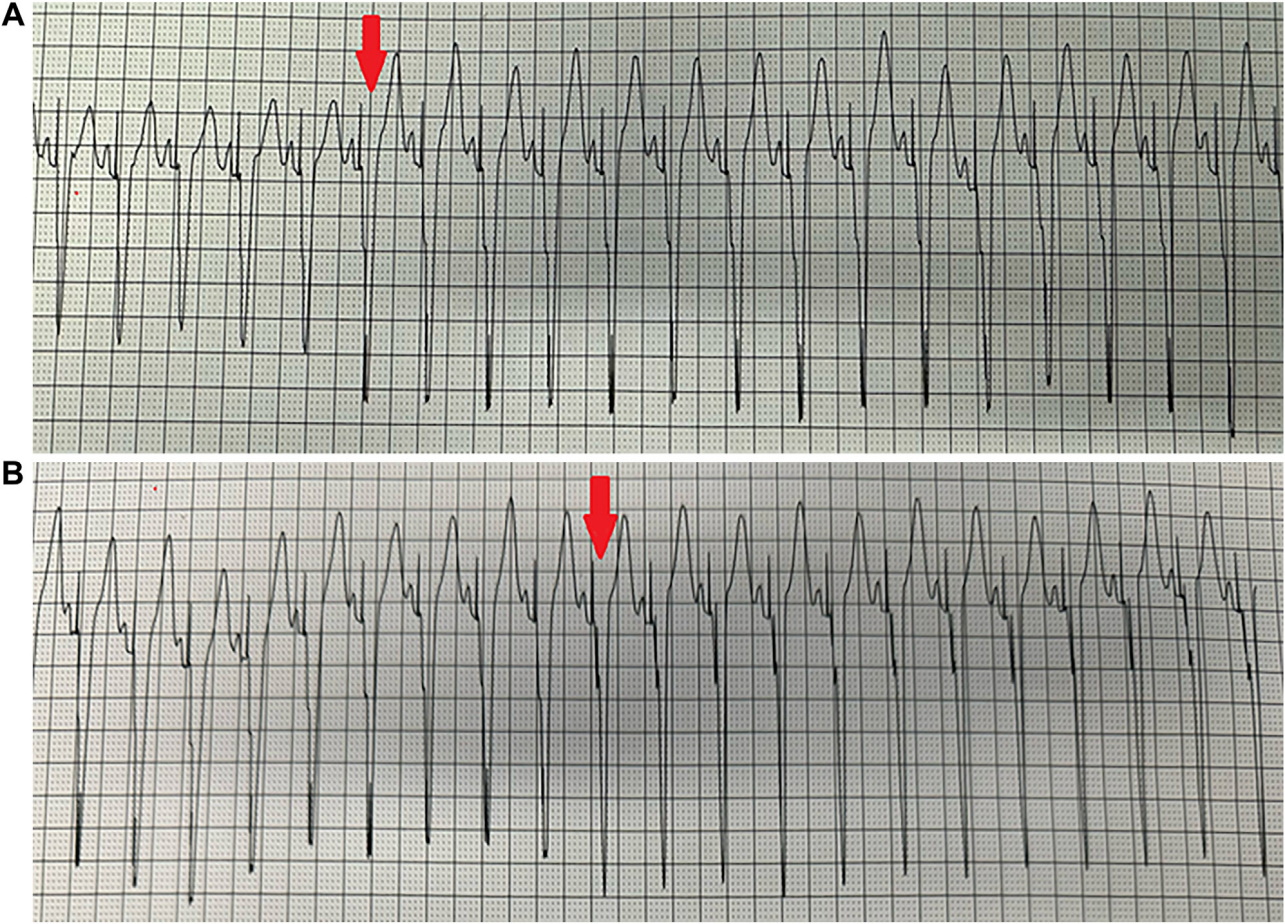
(A) Rhythm strip obtained during programming of the timing offset delay. Begins with timing delay of 0 ms, QRS duration of 125 ms, and changes to an 80-ms timing delay at the red arrow. After the 80 ms delay, the QRS duration jumps to 151 ms. (B) Rhythm strip obtained during programming of the timing offset delay. Begins with timing delay of 80 ms, QRS duration of 151 ms, and is changed to a 40-ms timing delay at the red arrow. After the 40-ms delay is established, the QRS complex narrows to 100 ms.

Previously considered a poor surgical candidate, the improved function allowed the subspecialist to address multiple comorbidities associated with DiGeorge syndrome during the remainder of the patient’s hospitalization. Discharge occurred 12 weeks post CRT therapy with normal cardiac function and no further cardiac interventions.

## Comment

Coordinated ventricular contraction is initiated via an intact His-Purkinje system. Uncoordinated electrical activation leads to uncoordinated ventricular contraction, resulting in the force generated by the early contracting segments to dissipate during relaxation of later contracting ventricular segments leading to depressed cardiac function and pathological remodeling.^1^

CRT uses implanted electrodes to coordinate the function between the left and right ventricles, improving cardiac output. Specific guidelines for CRT in the adult population exist, but no such guidelines exist in the congenital heart population.^2^

Much of the available CRT experience in children comes from congenital CHB. Song and associates^3^ reported long-term follow-up data for 34 patients with congenital CHB and no structural heart disease (median age at pacemaker insertion, 2.5 years). At a median follow-up of 12.3 years, 7 patients (20.6%) demonstrated LV dysfunction. Multivariate analysis revealed that right ventricular (RV) free wall pacing was the only independent risk factor for LV dysfunction in these patients.^3^ Janousek and colleagues^4^ reported that, in patients without structural heart disease, pacing the RV outflow tract or lateral RV predicts a depressed LV EF with an odds ratio of 10.7, while LV apical or midlateral LV wall pacing predicts preserved LV function.

Kubus and coworkers^5^ reported their experience with 32 patients who underwent CRT. A positive response was defined as an increase in the systemic ventricular EF >10% with the same or improved New York Heart Association class. They found that 55% of patients responded favorably, with patients with a systemic LV showing the greatest improvement.^5^

This experience leads to the question of the best initial methodology for initial pacemaker placement in patients with CHD, especially younger patients. Gebauer and associates^6^ evaluated LV function in 82 patients with CHB. LV dilation and dysfunction developed in 11 patients, and RV free wall pacing was the only significant risk factor (odds ratio, 14.3; *P* < .001). Authors concluded that “in the presence of a systemic LV, epicardial free wall pacing should, however, be avoided whenever possible” and that “free wall RV pacing contributes to dyssynchronous LV activation and potentially pathological LV remodeling and dysfunction.”^6^

According to CRT studies, 50% or more of CHD patients exhibit pacing-related ventricular dyssynchrony and heart failure.^7^

The experience with single-ventricle CRT is extremely limited but has shown promise. The “multisite pacing” technique reported by Cecchine and coworkers^7^ included 13 single-ventricle patients, 8 of whom had previously undergone pacemaker insertion for CHB. Eight of the 13 patients had improved New York Heart Association class or an increased EF of 10% or better. The overall positive response rate was 10 of 11 with longer-term follow-up.^7^

Pacing-induced ventricular dysfunction can occur in CHD patients with CHB. The location of pacemaker leads appears to be an important factor in the development of LV dysfunction. In CHD patients requiring a pacemaker, placement of an apical LV lead in lieu of a RV lead may reduce the likelihood of pacer-related ventricular dysfunction. CRT should be considered for paced postoperative CHB patients with LV dysfunction.
